# Evaluation of Coronary Artery Disease Plaques Using Coronary CT Angiography in a High-Risk Population

**DOI:** 10.7759/cureus.112206

**Published:** 2026-07-07

**Authors:** Angshumi Deka, Lipee Nath, Hemchandra Kalita, Saanjhi Rawat

**Affiliations:** 1 Radiodiagnosis, Assam Medical College and Hospital, Dibrugarh, IND; 2 Cardiology, Assam Medical College and Hospital, Dibrugarh, IND

**Keywords:** cad-rads, coronary artery calcium score, coronary artery disease, coronary atherosclerosis, coronary ct angiography, high-risk plaque, multivessel disease, stable chest pain, vulnerable plaque

## Abstract

Background

Coronary artery disease (CAD) remains a leading cause of cardiovascular morbidity and mortality. Coronary computed tomography angiography (CCTA) enables non-invasive assessment of coronary stenosis, plaque burden, and high-risk plaque (HRP) morphology, offering incremental risk information beyond traditional clinical scoring.

Objective

The objective of this study was to evaluate coronary plaque characteristics and the prevalence of HRP features on CCTA in high-risk patients presenting with stable chest pain at a tertiary care center in Assam, India, and to assess their association with the severity of coronary artery disease.

Methods

This hospital-based cross-sectional study included 40 patients with stable chest pain and major cardiovascular risk factors (type 2 diabetes mellitus, hypertension, dyslipidemia, smoking, and/or family history of CAD) referred for CCTA evaluation from November 2023 to October 2024. All patients underwent 128-slice CCTA with coronary artery calcium (CAC) scoring. Plaque presence, distribution, morphology, and HRP features - including low-attenuation plaque, napkin-ring sign, spotty calcification, and positive remodeling - were assessed along with the Coronary Artery Disease-Reporting and Data System (CAD-RADS) grading. Statistical analysis was performed using Fisher's exact test.

Results

CAD was detected in 28 of 40 patients (70%). High-risk (vulnerable) plaques were identified in 22 patients (55%), while stable calcified plaques were present in six patients (15%), and no plaque was detected in 12 patients (30%). Among HRP features, low-attenuation plaque was most common (n = 12, 30%), followed by napkin-ring sign (n = 7, 17.5%), spotty calcification (n = 2, 5%), and positive remodeling (n = 1, 2.5%). The left anterior descending artery was the most frequently involved vessel (n = 19, 47.5%). The majority of patients fell into the CAD-RADS 3 category (n = 15, 37.5%). A CAC score of 101-200 was the most prevalent range (42.5%). A statistically significant association was found between the number of cardiovascular risk factors and the presence of HRP (p = 0.033); however, no significant correlation was observed between the degree of coronary calcification and HRP features.

Conclusion

CCTA is a valuable non-invasive modality for detecting and characterizing coronary plaques in high-risk patients with stable chest pain. The high prevalence of HRP features, particularly in individuals with multiple cardiometabolic risk factors, underscores the role of CCTA in coronary risk stratification and guiding early preventive management.

## Introduction

Coronary artery disease (CAD) remains one of the leading causes of morbidity and mortality worldwide and develops through a prolonged interaction of modifiable and non-modifiable risk factors that promote atherosclerotic plaque formation within the coronary arteries [1,2]. As plaques evolve from stable lesions to vulnerable phenotypes prone to rupture, they may precipitate acute coronary syndromes, making early identification of high-risk plaque features a critical clinical objective [3-5].

Coronary computed tomography angiography (CCTA) has emerged as a robust non-invasive imaging modality capable of evaluating both the coronary lumen and vessel wall in a single examination, allowing simultaneous assessment of stenosis severity, overall plaque burden, and plaque composition [6-8]. Beyond the detection of calcified disease, CCTA can identify high-risk plaque characteristics such as low-attenuation plaque, positive remodeling, spotty calcification, and the napkin-ring sign, all of which have been consistently associated with plaque vulnerability and adverse cardiovascular outcomes [3,4,9-12].

In high-risk populations, including patients with diabetes mellitus, hypertension, and dyslipidemia, prior studies using CCTA have demonstrated a higher prevalence of non-calcified and high-risk plaque features, supporting the value of detailed plaque characterization in addition to stenosis assessment [13-15]. In such patients presenting with stable chest pain at tertiary care centers, anatomical and plaque-level evaluation with CCTA may provide incremental risk information beyond traditional clinical risk scores or coronary artery calcium scoring alone [7,11,14].

Despite the well-established burden of CAD in Northeast India - where CAD prevalence is estimated at approximately 7.4% in Assam, lower than the national average of 10.5% but still substantial in absolute terms - data on CCTA-based plaque characterization and high-risk plaque (HRP) feature prevalence from this region remain sparse. The present study, therefore, aims to evaluate coronary plaque characteristics and the prevalence of HRP features on CCTA in patients with stable chest pain and cardiovascular risk factors at a tertiary care center in Assam, and to assess the association between cardiovascular risk factor burden and the presence of high-risk plaque morphology, with a view to informing regional risk stratification strategies.

## Materials and methods

Study design and setting

This hospital-based cross-sectional study was conducted in the Department of Radiodiagnosis, Assam Medical College and Hospital, Dibrugarh, Assam, India, over a period of one year from November 2023 to October 2024. Patients were referred from the Department of Cardiology for coronary computed tomography angiography (CCTA) evaluation.

Study population and sample size

The study population comprised patients with high-risk features presenting with stable chest pain who were sent for CCTA from cardiology. In this study, "stable chest pain" was defined as chest pain that was clinically stable in character - reproducible, non-accelerating, and present for at least two weeks without change in frequency, severity, or precipitating threshold. Patients with pain occurring exclusively and transiently at rest were not considered stable and were further evaluated by the referring cardiologist for acute coronary syndrome or vasospastic angina prior to any CCTA referral. All enrolled patients had a normal or non-diagnostic ECG at presentation and did not fulfill clinical or biomarker criteria for ACS, with normal serum troponin and absence of ST-segment changes. Vasospastic angina was considered and excluded by the referring cardiologist based on symptom pattern - specifically the absence of rest-only pain, the presence of effort-related or mixed pattern symptoms, and the absence of ST-segment changes during symptomatic episodes - before referral for CCTA. Taking the prevalence of non-calcified plaque as 11.1% and an absolute error of 10%, the calculated sample size was 39.47, which was rounded to 40 at 80% power and 95% confidence interval.

Inclusion and exclusion criteria

Eligible participants were adults with stable chest pain and at least one high-risk feature: diabetes mellitus without target-organ damage of more than 10 years' duration, or shorter-duration diabetes when combined with other risk factors such as hypertension or smoking; total cholesterol ≥250 mg/dL or LDL ≥130 mg/dL; a family history of major cardiovascular events with or without other risk factors; or young patients with type 1 diabetes mellitus ≤35 years or type 2 diabetes mellitus ≤50 years. Patients with documented atherosclerotic cardiovascular disease, including prior acute coronary syndrome (ACS) or myocardial infarction (MI), unstable or stable angina, coronary revascularization, stroke or transient ischemic attack (TIA), those refusing consent, and those with contrast allergy, pregnancy, or chronic kidney disease were excluded.

CT protocol and image acquisition

All CCTA examinations were performed on a 128-slice Revolution EVO CT scanner (GE Healthcare, Chicago, Illinois, United States) with patients positioned supine, arms raised, and ECG leads attached for prospective ECG-gated acquisition, usually targeting mid-diastole at heart rates ≤70 beats per minute. Heart rate and blood pressure were recorded before scanning; when heart rate exceeded 70 beats per minute, an oral beta-blocker (propranolol 20-40 mg about one hour before the scan) was given as needed, after advising patients to fast for three to four hours, avoid caffeine for 12 hours, and practice breath-holding.​

The scan range extended from just above the tracheal bifurcation to below the cardiac apex in a craniocaudal direction. After planning scans, approximately 80 mL of non-ionic iodinated contrast (e.g., iohexol or iopromide) was injected through an antecubital vein at 5 mL/s, followed by a 40 mL saline chaser, and images were acquired during inspiratory breath-hold.

Image reconstruction and analysis

Data were reconstructed as submillimetre ECG-gated datasets and evaluated using axial, coronal, and sagittal planes, along with multiplanar reformation (MPR), curved MPR (cMPR), maximum intensity projection (MIP), and volume rendering techniques (VRT), with optimized window settings and field of view. Coronary segments were assessed for the presence and extent of plaque, plaque type (calcified, non-calcified, mixed), and high-risk features including low-attenuation plaque (typically less than 30 HU, which reflects a large lipid-rich necrotic core), napkin-ring sign (a low-attenuation core encircled by a higher-attenuation rim), spotty calcification (small, focal calcific deposits within non-calcified plaque), and positive remodeling (indicates outward vessel expansion at the plaque site). Stenosis severity and overall disease burden were graded using the Coronary Artery Disease Reporting and Data System (CAD-RADS) classification system [16]. All CCTA examinations were interpreted by an experienced radiologist with dedicated cardiac CT training. Cases with complex or borderline plaque morphology were reviewed in conjunction with the cardiology co-investigator to reach a consensus interpretation. Formal inter-observer variability assessment using kappa statistics or intraclass correlation coefficients was not performed as a pre-specified study endpoint and is acknowledged as a limitation.

Statistical methods

Data were entered into Excel 2010 (Microsoft, Redmond, Washington, United States) and analyzed using SPSS version 20.0 (IBM Inc., Armonk, New York, United States). Categorical variables were summarized as frequencies and percentages, and associations between high-risk factors and the presence of vulnerable plaque were tested using Fisher's exact test, with a p-value < 0.05 considered statistically significant. 

## Results

The baseline demographic and clinical characteristics of the study population are summarized in Table 1. The cohort comprised 40 patients with a mean age of 61.38 ± 13.45 years (range 34-85 years), with males constituting 70% (n = 28) and females 30% (n = 12). The most prevalent cardiovascular risk factor was diabetes mellitus (50%, n = 20), followed by hypertension (45%, n = 18), smoking (35%, n = 14), family history of CAD (32.5%, n = 13), and dyslipidemia (20%, n = 8). The majority of patients (52.5%, n = 21) harbored more than one cardiovascular risk factor. Patients with chronic kidney disease were excluded prior to enrollment in accordance with standard CCTA contrast safety protocols, as contrast nephropathy in this subgroup poses an unacceptable procedural risk

**Table 1:** Baseline characteristics table All patients with chronic kidney disease were excluded prior to enrollment in accordance with standard CCTA contrast safety protocols. CKD exclusion was applied at the clinical referral stage. CAD - coronary artery disease; CCTA - coronary computed tomography angiography; CKD - chronic kidney disease

Characteristic	Value
Age, mean ± SD (years)	61.38 ± 13.45
Age range (years)	34–85
Male sex, n (%)	28 (70.0)
Female sex, n (%)	12 (30.0)
Male-to-female ratio	2.33:1
Cardiovascular risk factors	
Diabetes mellitus, n (%)	20 (50.0)
Hypertension, n (%)	18 (45.0)
Smoking, n (%)	14 (35.0)
Family history of CAD, n (%)	13 (32.5)
Dyslipidemia, n (%)	8 (20.0)
Renal function	
Chronic kidney disease	Excluded per protocol

Type of plaque

**Figure 1:**
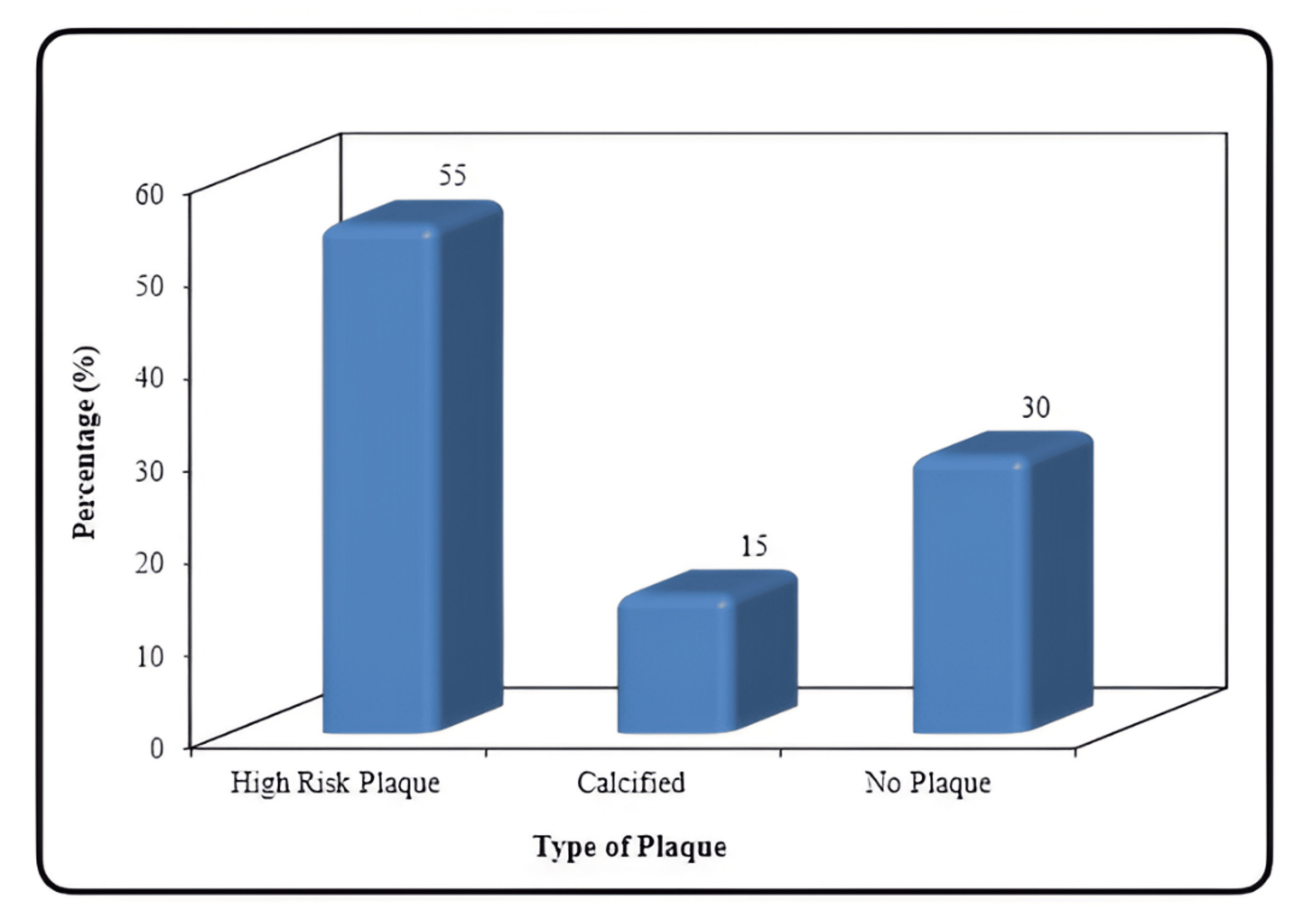
Type of plaque

The data (Table 2, Figure 1) indicates that 55% (n=22) of the study population had high-risk plaques, which are more prone to rupture and may lead to serious cardiovascular events. Fifteen percent (n=6) had calcified plaques, which are generally more stable but can still contribute to artery narrowing and restricted blood flow. Thirty percent (n=12) had no detectable plaque, suggesting a lower risk of coronary artery disease.

**Table 2:** Type of plaque

Type of plaque	Number (n)	Percentage (%)
High risk plaque	22	55.00
Calcified	6	15.00
No plaque	12	30.00
Total	40	100.00

Distribution of atherosclerotic plaque in coronary arteries

**Figure 2:**
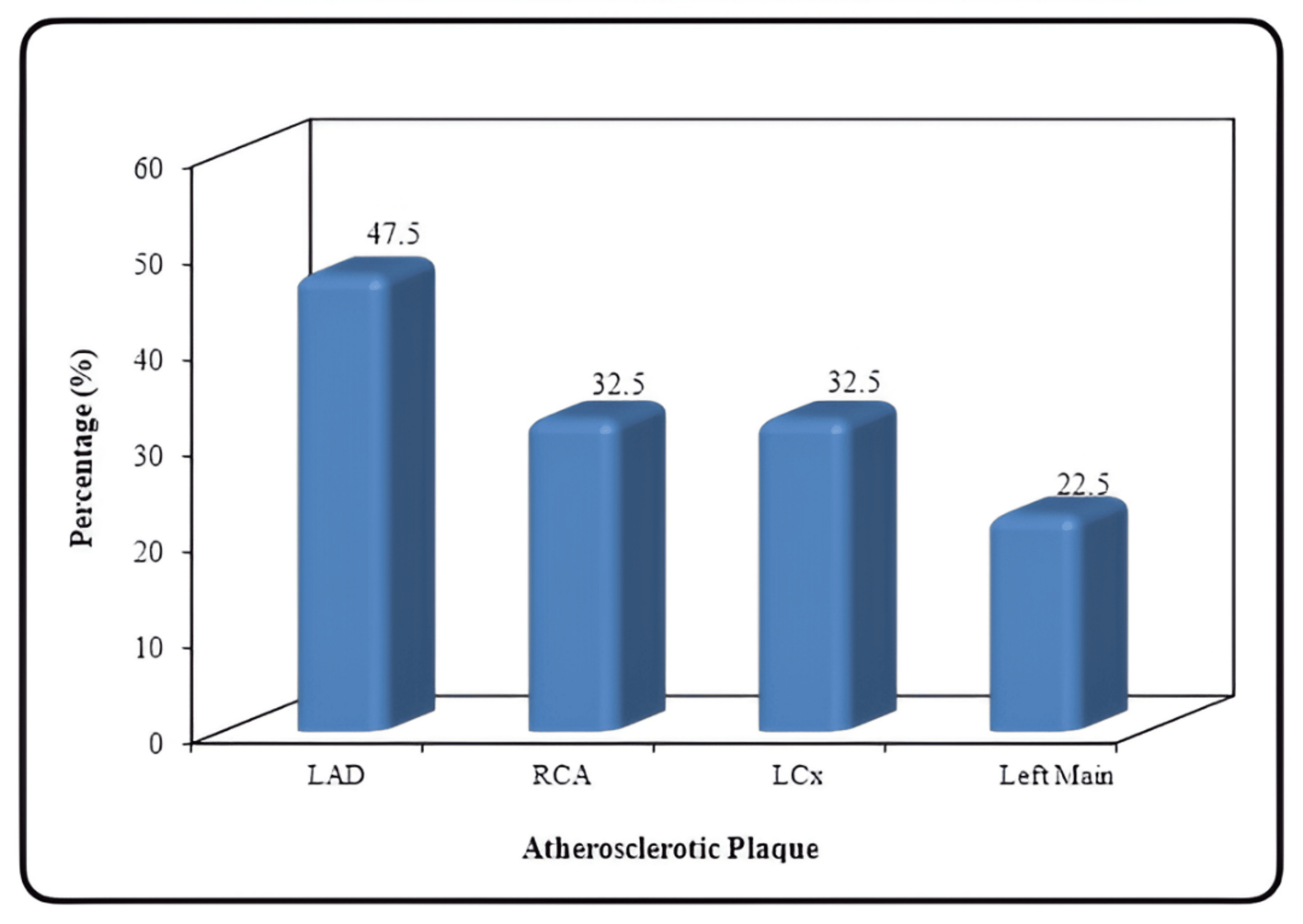
Distribution of plaque in coronary arteries LAD - left anterior descending; RCA - right coronary artery; LCx - left circumflex artery

The data (Table 3, Figure 2) shows the distribution of atherosclerotic plaque in different coronary arteries among the study population. The left anterior descending (LAD) artery is the most commonly affected, with 47.5% (n=19) having plaque, highlighting its vulnerability to atherosclerosis and its critical role in myocardial blood supply. The right coronary artery (RCA) and left circumflex artery (LCx) are equally affected in 32.5% (n=13) of cases, indicating a significant burden of disease in these vessels. The left main coronary artery, supplying both the LAD and LCx, is affected in 22.5% (n=9), representing a high-risk subgroup requiring careful monitoring and intervention.

**Table 3:** Distribution of atherosclerotic plaque in coronary arteries Percentages are calculated per patient using the total sample size (n=40). As more than one coronary artery may be involved in the same patient, cumulative percentages may exceed 100%. LAD - left anterior descending; RCA - right coronary artery; LCx - left circumflex artery

Atherosclerotic plaque	Number	Percentage (%)
LAD	19	47.50
RCA	13	32.50
LCx	13	32.50
Left main	9	22.50

Plaque characteristics

**Figure 3:**
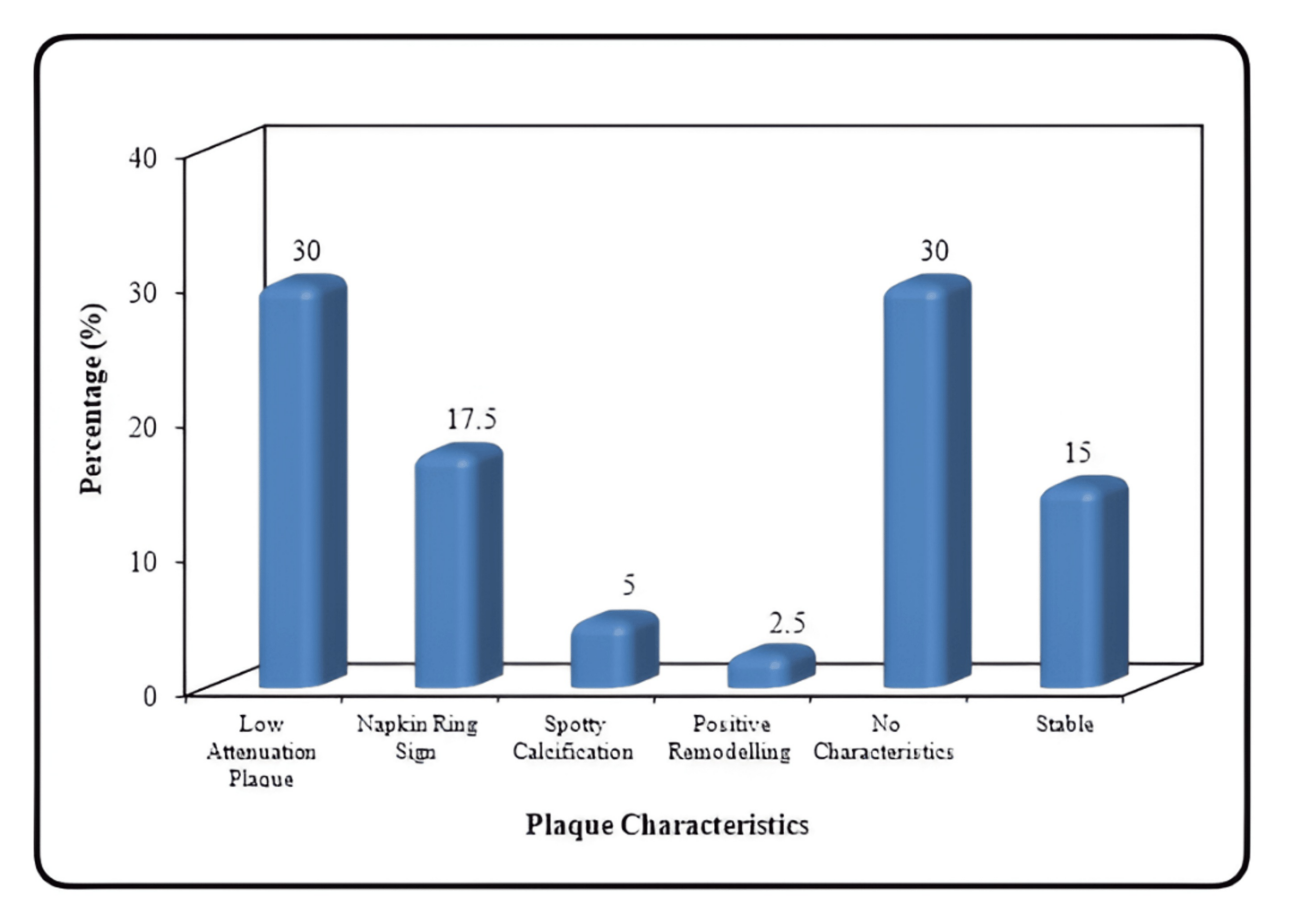
Plaque characteristics

Our data categorizes (Table 4, Figure 3) plaque characteristics in the study population. Low-attenuation plaque (n = 12, 30%) is the most common high-risk feature, associated with vulnerability to rupture. The napkin-ring sign (n = 7, 17.5%), a strong predictor of plaque instability, is present in a smaller subset. Spotty calcification (n = 2, 5%) and positive remodeling (n = 1, 2.5%) indicate early or progressive atherosclerosis. Notably, 30% (n = 12) of patients had no high-risk plaque characteristics, while 15% (n = 6) had stable plaques, suggesting a lower risk of acute events.

**Table 4:** Plaque characteristics

Plaque characteristics	Number (n)	Percentage (%)
Low attenuation plaque	12	30.00
Napkin ring sign	7	17.50
Spotty calcification	2	5.00
Positive remodelling	1	2.50
No characteristics	12	30.00
Stable	6	15.00
Total	40	100.00

CAD-RADS scoring

**Figure 4:**
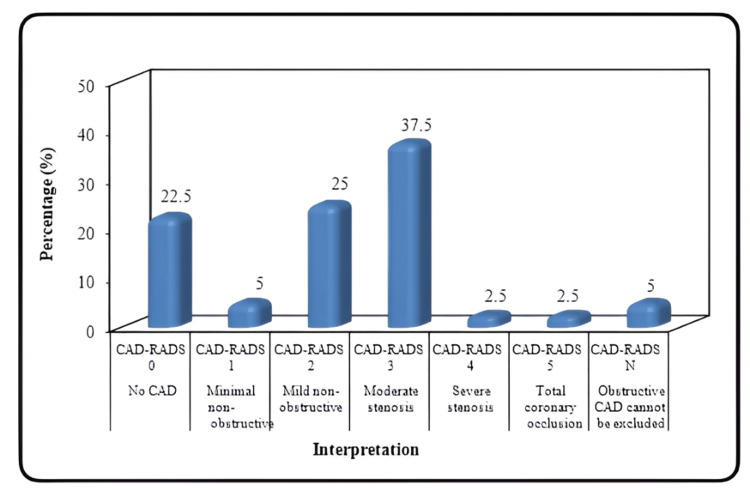
CAD-RADS scoring CAD - coronary artery disease; CAD-RADS - Coronary Artery Disease-Reporting and Data System

The data (Table 5, Figure 4) classifies coronary artery disease (CAD) severity using the CAD-RADS system. 22.5% (n=9) had no detectable CAD (CAD-RADS 0), while 30% (n=12) had minimal to mild non-obstructive disease (CAD-RADS 1-2), indicating early- stage atherosclerosis. 37.5% (n=15) had moderate stenosis (CAD-RADS 3), suggesting a significant yet non-critical narrowing. Severe stenosis (CAD-RADS 4, n = 1, 2.5%) and total occlusion (CAD-RADS 5, n = 1, 2.5%) were less common but represent high-risk cases requiring intervention. In 5% (n=2), obstructive CAD could not be ruled out (CAD-RADS N).

**Table 5:** CAD-RADS scoring CAD - coronary artery disease; CAD-RADS - Coronary Artery Disease-Reporting and Data System

Interpretation	Classification	Number (n)	Percentage (%)
No CAD	CAD-RADS 0	9	22.50
Minimal non-obstructive	CAD-RADS 1	2	5.00
Mild non-obstructive	CAD-RADS 2	10	25.00
Moderate stenosis	CAD-RADS 3	15	37.50
Severe stenosis	CAD-RADS 4	1	2.50
Total coronary occlusion	CAD-RADS 5	1	2.50
Obstructive CAD cannot be excluded	CAD-RADS N	2	5.00
Total		40	100.00

Statistical analysis of plaque with risk factors

**Figure 5:**
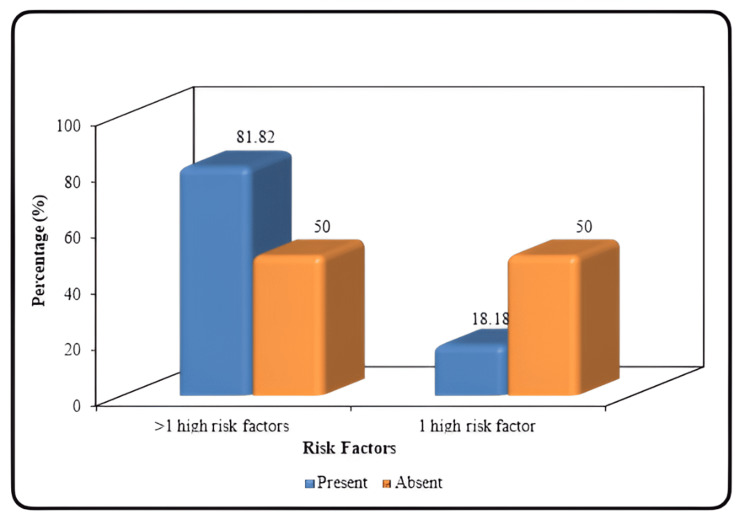
Statistical analysis of vulnerable plaque with risk factors

The data (Table 6, Figure 5) examines the relationship between risk factors (such as diabetes mellitus, hypertension, smoking, family history of hypertension, and dyslipidemia) and the presence of vulnerable plaque. Among individuals with more than one high-risk factor, 81.82% (n=18) had plaque, while only 50% (n=9) were plaque-free. In contrast, among those with only one high-risk factor, 18.18% (n=4) had plaque, while 50% (n=9) did not. The p-value (0.033) indicates a statistically significant association, suggesting that individuals with multiple risk factors are more likely to develop vulnerable plaque.

**Table 6:** Statistical analysis of vulnerable plaque with risk factors * Fisher's exact test; the p-value is significant at 5% level of significance

Risk factors	Vulnerable plaque				p-value
	Present		Absent		
	n	%	n	%	
>1 high-risk factors	18	81.82	9	50.00	0.033*
1 high-risk factor	4	18.18	9	50.00	
Total	22	100.00	18	100.00	

## Discussion

In this study, we observed a substantial burden of coronary atherosclerosis among high-risk patients presenting with stable chest pain in Northeast India. What is particularly striking is not just the presence of plaque, but the frequency of high-risk plaque (HRP) characteristics, especially non-calcified and mixed plaques. These findings align closely with the SCOT-HEART trial, which demonstrated that adverse plaque features on CCTA predict future coronary events independently of stenosis severity [1]. Similarly, analysis from the PROMISE trial confirmed that identifying HRP features adds meaningful prognostic information in stable chest pain populations [3]. Together, these landmark studies, along with contemporary imaging perspectives [2], reinforce a key message also reflected in our cohort: coronary risk is not defined solely by how narrow a vessel appears, but by how biologically vulnerable the plaque itself may be.

**Figure 6:**
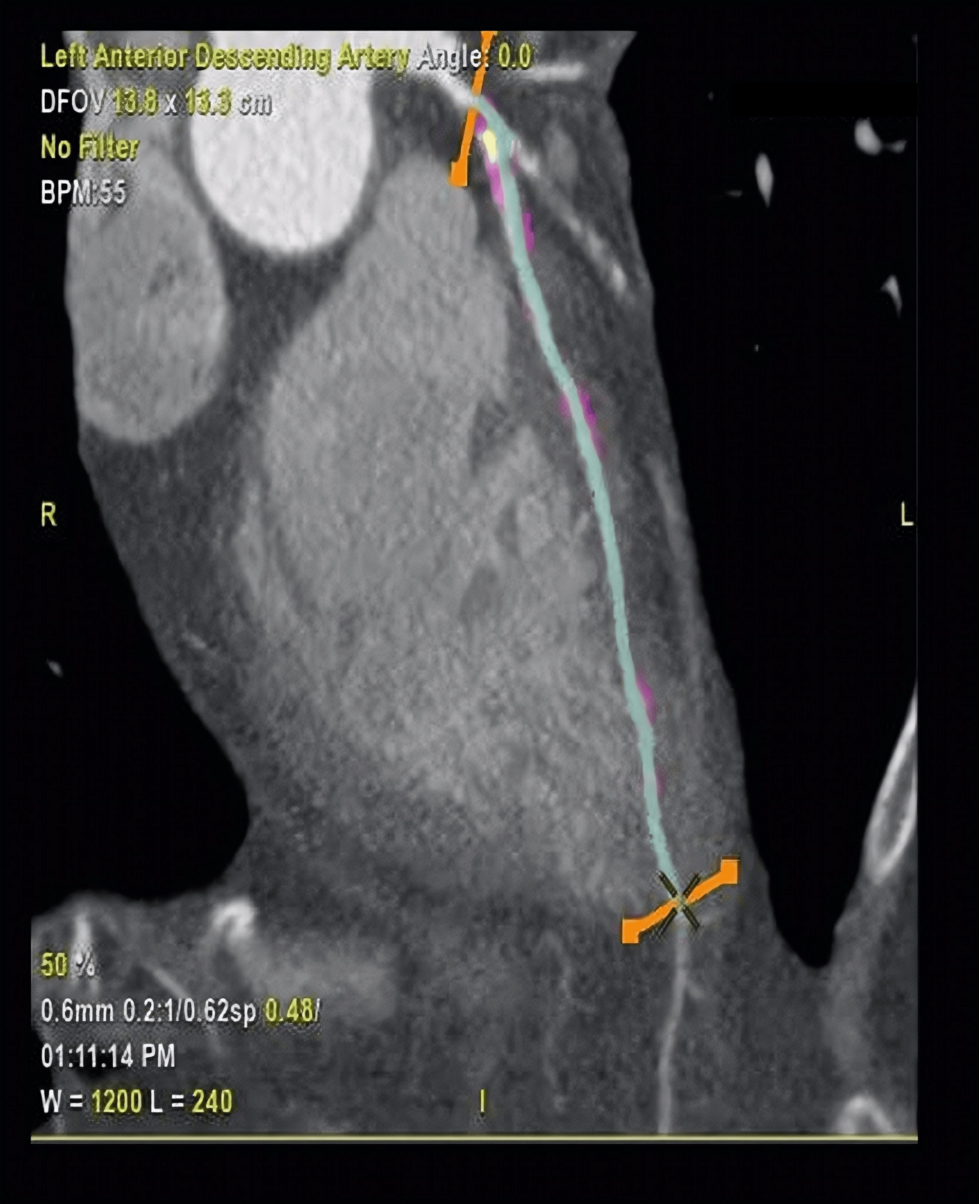
High risk plaque with spotty calcification Curved axial view showing a high-risk plaque with spotty calcification (color-coded in yellow) within the left main artery (lumen color-coded in teal), extending to the left anterior descending artery and the left circumflex artery. This image is original and derived from the present study.

**Figure 7:**
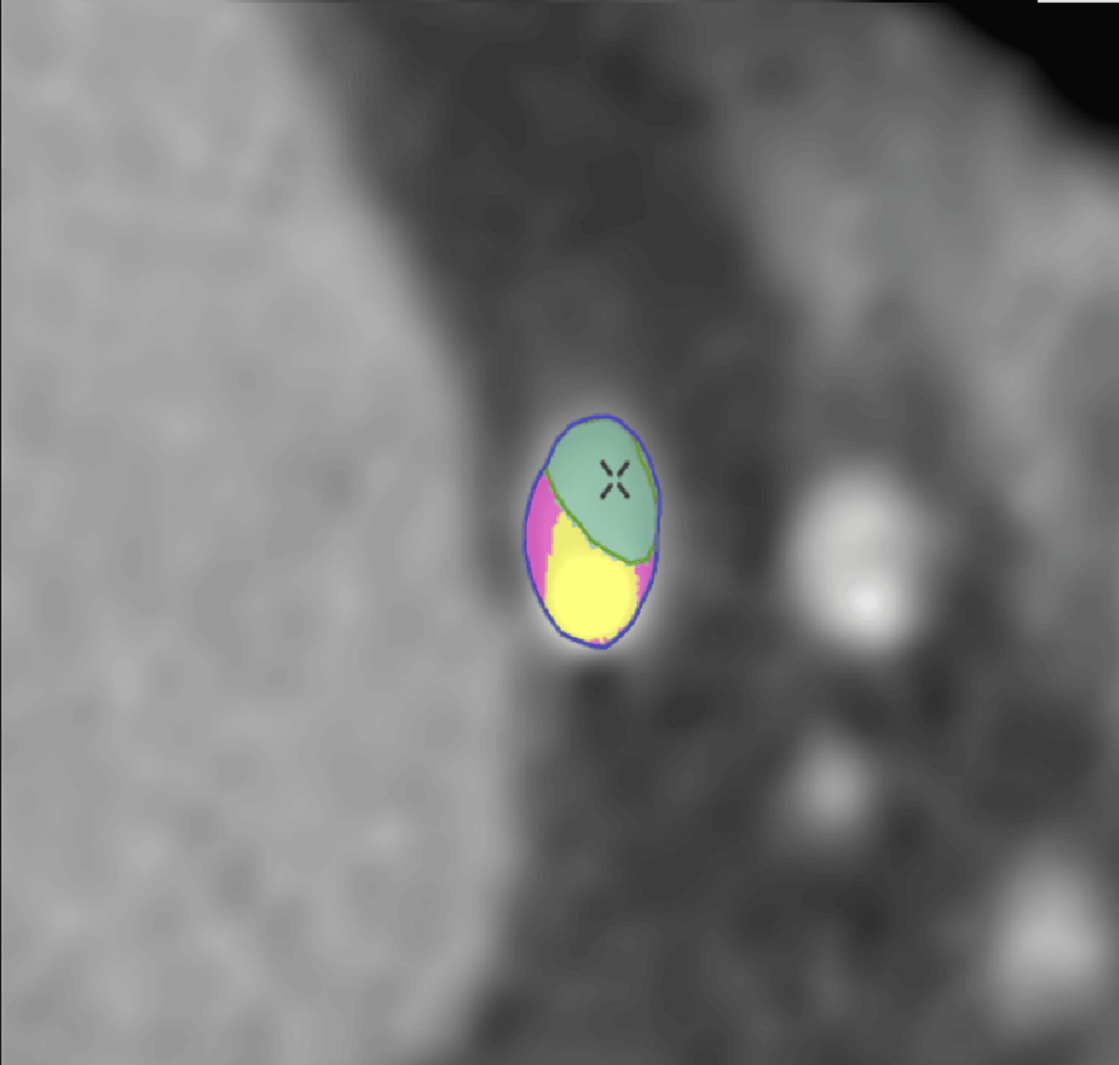
High risk plaque with spotty calcification High-risk plaque with spotty calcification. Luminal view showing a high-risk plaque with spotty calcification (in yellow) within a soft plaque (in pink) in the left main artery. This image is original and derived from the present study.

**Figure 8:**
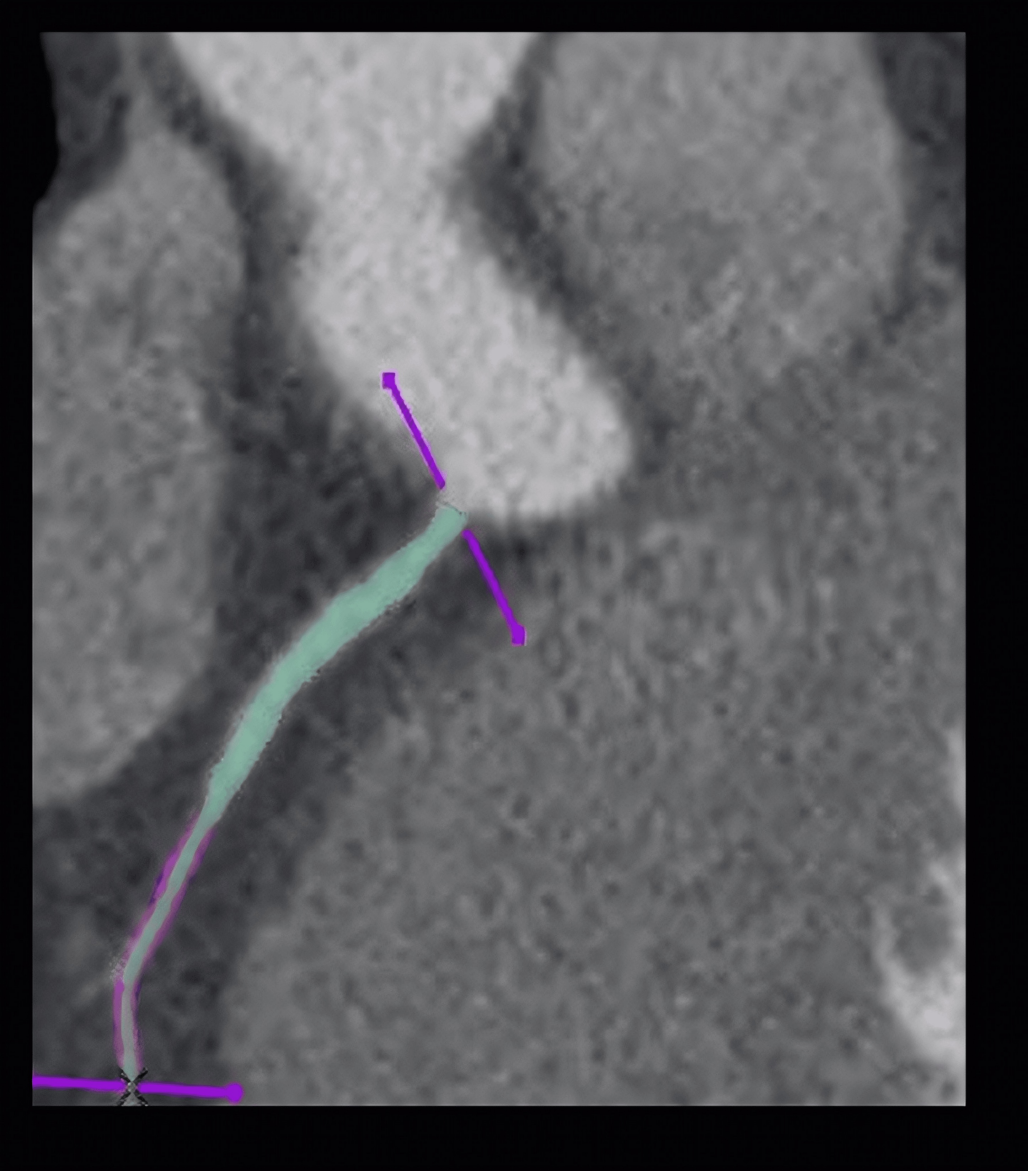
Curved axial view showing low attenuation plaque in right coronary artery not causing significant luminal narrowing A segment of the right coronary artery has been traced from its origin (indicated by purple arrows). The low-attenuation plaque that can be noted in the distal traced portion is color-coded in pink color, and the right coronary artery lumen is delineated in teal color; no significant luminal narrowing is identified. This image is original and derived from the present study.

More than half of our patients exhibited HRP features like low attenuation plaque, napkin-ring sign and spotty calcification (Figure 6 and Figure 7), with low-attenuation plaque (Figure 8) and the napkin-ring sign being the most frequent. Prior longitudinal data have shown that these particular features, especially low-attenuation plaque (<60 HU) and napkin-ring sign, are among the strongest CT predictors of major adverse cardiac events [8]. Importantly, the ROMICAT-II trial demonstrated that HRP can predict acute coronary syndromes even in the absence of obstructive stenosis [4], and similar associations have been reported for non-calcified vulnerable plaques in long-term follow-up studies [13]. Our findings echo this evidence and emphasize a clinically important concept: plaques that appear only moderately stenotic may still carry significant biological risk.

The predominance of LAD involvement in our cohort is also clinically meaningful (Figure 6). Large registries examining precursors of acute coronary syndromes have consistently shown that LAD plaques are frequently implicated in culprit events [12]. The pattern seen in our patients mirrors these observations and reinforces the central role of LAD disease in adverse outcomes. Furthermore, the predominance of CAD-RADS 3 and CAD-RADS 2 categories suggests that many patients fall into a therapeutic "gray zone", where aggressive medical management and close surveillance may be more appropriate than immediate revascularization. Evidence from the Collaborative Meta-Analysis of Cardiac CT (CoMe-CCT) Consortium supports the diagnostic and management value of CCTA in such stable chest pain populations [6], and regional studies have demonstrated meaningful correlations between CCTA findings and clinical risk scores [14].

One of the most important observations in our study is the strong association between cardiometabolic risk factors - particularly type 2 diabetes mellitus - and extensive non-calcified and mixed plaque burden. Previous CCTA studies have shown that diabetic patients harbor more extensive plaque and derive incremental prognostic information from CCTA beyond calcium scoring alone [9,10]. More recent work has demonstrated that dyslipidemia and hypertension exert additive adverse effects in diabetics, promoting greater plaque burden and vulnerability [11,12]. Similarly, patients with metabolic syndrome have been shown to exhibit a higher prevalence of HRP features and worse outcomes [7]. The patterns observed in our cohort are therefore not isolated findings; rather, they reflect a broader and concerning trend of accelerated and potentially aggressive plaque phenotypes in metabolically high-risk populations.

Another noteworthy aspect of our study is the detection of HRP features in individuals without previously documented atherosclerotic cardiovascular disease. This finding carries practical implications. In SCOT-HEART, the incorporation of CCTA into routine care was associated with a reduction in myocardial infarction rates, likely through earlier identification of high-risk plaque and subsequent intensification of preventive therapy [1]. Our findings support this preventive paradigm: identifying vulnerable plaque before it becomes flow-limiting may offer a critical window for intervention.

**Figure 9:**
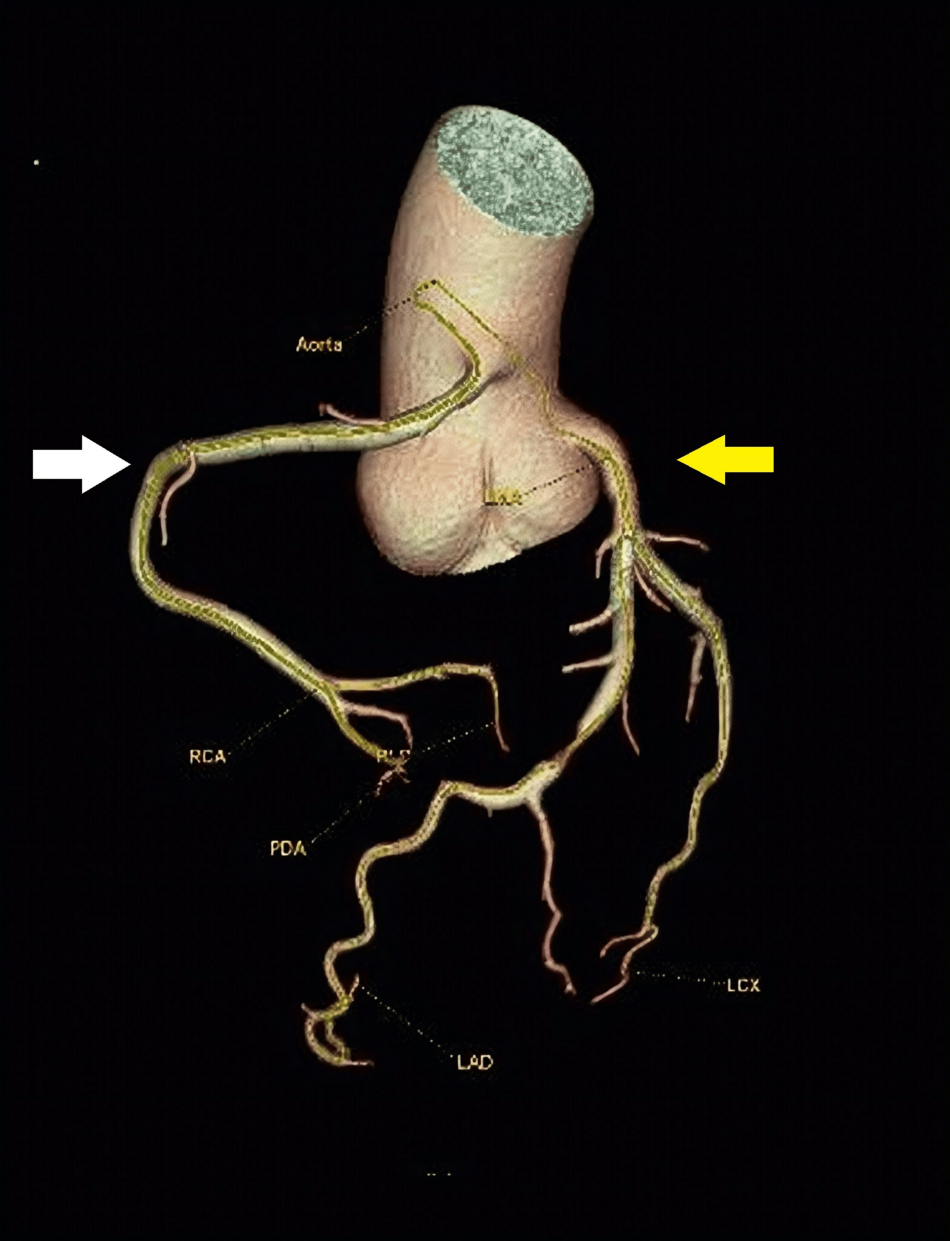
Anatomical variation in origin of right coronary artery Volume-rendered image showing the right coronary artery (white arrow) arising high above the right coronary sinus (approximately 1.8 cm from the sinotubular junction, indicated by the black line), with the origin of the left coronary artery (yellow arrow) from the usual left coronary sinus. This image is original and derived from the present study.

**Figure 10:**
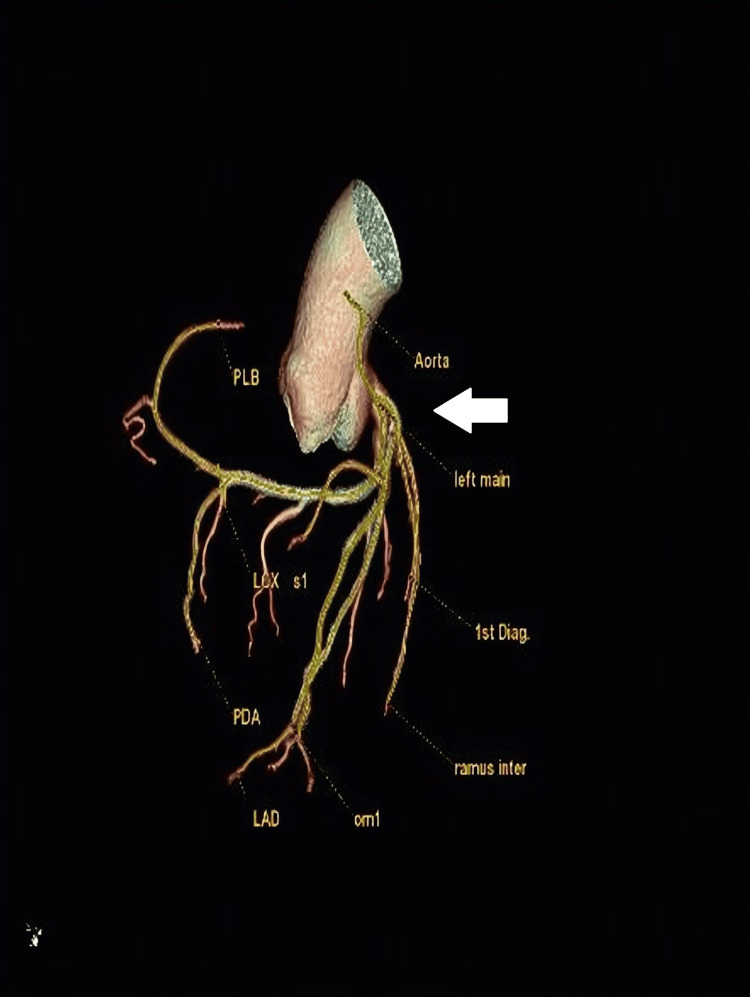
Congenital absence of right coronary artery 3-D reconstruction of coronary arteries showing the absence of the right coronary artery and the presence of only left coronary circulation (white arrow) supplying the entire heart. This image is original and derived from the present study.

Beyond plaque characterization, CCTA in our cohort also revealed congenital coronary anomalies, including anomalous origin of the right coronary artery (Figure 9) and single left coronary circulation (Figure 10). These findings highlight an additional strength of CCTA - the ability to comprehensively evaluate both coronary anatomy and plaque morphology in a single, non-invasive examination. Such an integrated assessment has implications not only for risk prediction but also for procedural planning and long-term management.

Taken together, our results underscore the importance of moving beyond simple stenosis grading in high-risk South Asian patients with stable chest pain. Systematic plaque characterization and structured CAD-RADS reporting provide deeper insight into disease biology and patient-specific risk. In populations with a high burden of diabetes, hypertension, and dyslipidemia, early identification of vulnerable plaque - particularly low-attenuation plaque, napkin-ring sign, and spotty calcification - offers a tangible opportunity to intensify preventive therapy. In this context, CCTA emerges not merely as a diagnostic modality but as a powerful tool for personalized cardiovascular risk stratification and early intervention.

The clinical implications of identifying HRP features in this population extend beyond diagnostic labeling. Evidence from the SCOT-HEART trial demonstrated that incorporation of CCTA into routine care was associated with a reduction in myocardial infarction rates, likely through earlier identification of high-risk plaque and subsequent intensification of preventive therapy [1]. In practical terms, identification of low-attenuation plaque or napkin-ring sign on CCTA in a high-risk patient with stable chest pain should prompt reassessment of statin intensity - with consideration of high-intensity statin therapy if not already prescribed - along with optimization of blood pressure and glycemic control, evaluation for antiplatelet therapy where appropriate, and more frequent cardiological surveillance. This is particularly relevant in the Northeast Indian population, where a high burden of diabetes, hypertension, and dyslipidemia coexists with relatively limited access to advanced cardiovascular monitoring. By providing simultaneous assessment of coronary anatomy, stenosis severity, plaque burden, and morphological vulnerability in a single non-invasive examination, CCTA offers a uniquely comprehensive platform for individualized cardiovascular risk stratification in this population.

Limitations

First, the sample size of 40 patients from a single tertiary care center limits statistical power and the ability to draw definitive conclusions; the results should therefore be considered hypothesis-generating and requiring validation in larger multicenter cohorts. Second, the single-center design may limit generalizability to centers with different patient demographics, referral patterns, and risk factor profiles. Third, the cross-sectional study design precludes causal inference and does not allow determination of whether the HRP features identified on CCTA subsequently translate into adverse cardiovascular events. Longitudinal follow-up of this cohort is planned as future work. Fourth, medication data - including statin use, antiplatelet therapy, and antidiabetic agents - were not systematically collected, and the potential confounding effect of these medications on plaque morphology and cardiovascular risk cannot be excluded. Fifth, patients with chronic kidney disease were excluded per contrast safety protocols, which limits the applicability of these findings to a population known to harbor accelerated and extensive coronary atherosclerosis. And sixth, formal inter-observer reliability analysis for plaque characterization was not performed as a pre-specified study endpoint; all scans were read by a single experienced cardiac radiologist, with consensus review of borderline cases by the cardiology co-investigator.

## Conclusions

Coronary artery disease remains a major contributor to morbidity and mortality, and the present study demonstrates that a substantial proportion of high-risk patients with stable chest pain in Northeast India already harbor significant coronary atherosclerosis on CCTA, including plaques with high-risk morphological features. Importantly, non-calcified and mixed plaques, along with characteristics such as low-attenuation plaque, napkin-ring sign, spotty calcification, and positive remodeling, were frequently observed even in individuals without previously documented atherosclerotic cardiovascular disease. These findings underscore the aggressive and often clinically silent nature of disease in this population and emphasize the need to move beyond stenosis severity alone when interpreting coronary imaging.

By integrating assessment of luminal narrowing, plaque composition, high-risk features, and CAD-RADS classification, CCTA provided refined imaging-based risk stratification beyond traditional clinical risk factors and identified actionable markers to guide tailored medical therapy and closer follow-up. Selective application of CCTA in high-risk patients with stable chest pain may therefore enhance early detection of vulnerable plaque, support more informed clinical decision-making, and potentially improve long-term cardiovascular outcomes.
